# Multiscale composite model of fiber-reinforced tissues with direct representation of sub-tissue properties

**DOI:** 10.1007/s10237-019-01246-x

**Published:** 2019-11-04

**Authors:** Minhao Zhou, Semih E. Bezci, Grace D. O’Connell

**Affiliations:** 1 Department of Mechanical Engineering, University of California, Berkeley, 5122 Etcheverry Hall, #1740, Berkeley, CA 94720-1740 USA; 2 Department of Orthopaedic Surgery, University of California, San Francisco, San Francisco, USA

**Keywords:** Finite element modeling, Multiscale modeling, Structure-based, Structure–function relationship, Annulus fibrosus

## Abstract

In many fiber-reinforced tissues, collagen fibers are embedded within a glycosaminoglycan-rich extrafibrillar matrix. Knowledge of the structure–function relationship between the sub-tissue properties and bulk tissue mechanics is important for understanding tissue failure mechanics and developing biological repair strategies. Difficulties in directly measuring sub-tissue properties led to a growing interest in employing finite element modeling approaches. However, most models are homogeneous and are therefore not sufficient for investigating multiscale tissue mechanics, such as stress distributions between sub-tissue structures. To address this limitation, we developed a structure-based model informed by the native annulus fibrosus structure, where fibers and the matrix were described as distinct materials occupying separate volumes. A multiscale framework was applied such that the model was calibrated at the sub-tissue scale using single-lamellar uniaxial mechanical test data, while validated at the bulk scale by predicting tissue multiaxial mechanics for uniaxial tension, biaxial tension, and simple shear (13 cases). Structure-based model validation results were compared to experimental observations and homogeneous models. While homogeneous models only accurately predicted bulk tissue mechanics for one case, structure-based models accurately predicted bulk tissue mechanics for 12 of 13 cases, demonstrating accuracy and robustness. Additionally, six of eight structure-based model parameters were directly linked to tissue physical properties, further broadening its future applicability. In conclusion, the structure-based model provides a powerful multiscale modeling approach for simultaneously investigating the structure–function relationship at the sub-tissue and bulk tissue scale, which is important for studying multiscale tissue mechanics with degeneration, disease, or injury.

**Electronic supplementary material:**

The online version of this article (10.1007/s10237-019-01246-x) contains supplementary material, which is available to authorized users.

## Introduction

Many soft tissues in the body include highly aligned collagen fibers embedded in a glycosaminoglycan-rich extrafibrillar matrix. The matrix allows for water and nutrient absorption, which is important for maintaining tissue homeostasis (Yang and O’Connell 2019), while fibers create anisotropic mechanical properties that allow the tissue to withstand large tensile loads. For example, tendons and ligaments have a single family of fibers, providing the tissue with greater stiffness along the primary in situ loading direction (Benjamin and Ralphs 1997). Meanwhile, tissues that undergo multiaxial loadings have more complex fiber networks, from two fiber populations, such as arterial walls and the annulus fibrosus (AF) of the intervertebral disk (Holzapfel et al. 2000; Adams and Roughley 2006), to randomly distributed fibers, such as skin (Cotta-Pereira et al. 1976).

Structural and mechanical behaviors of fibers and the matrix have been shown to change with degeneration, disease, and injury. For example, the AF has a cross-ply fiber structure (Cassidy et al. 1989; Marchand and Ahmed 1990), where collagen fibers can reorient under tensile loading. The amount of fiber reorientation has been shown to decrease with degeneration (Guerin and Elliott 2006), partly due to matrix stiffening and increased collagen cross-linking (Fujita et al. 1997; Wagner et al. 2006; O’Connell et al. 2011), which can lead to increased stress concentrations within the disk, triggering catabolic remodeling that can cause tissue failures (Antoniou et al. 1996; Adams and Roughley 2006). Failure of these fiber-reinforced tissues can cause a wide range of clinical issues, from mechanical dysfunctions of the disk to death (e.g., a ruptured aneurysm) (Juvela et al. 2000; Rubin 2007; Erwin and Hood 2014; O’Connell et al. 2015). Therefore, it is important to understand the role sub-tissue properties (e.g., fiber networks, matrix biochemical compositions, etc.) play on bulk tissue mechanics.

Although experimental studies have provided important information regarding bulk tissue mechanics, there are few studies that have directly measured sub-tissue properties due to challenges in conducting tests on individual tissue subcomponents. Thus, many researchers have complemented experimental data with structure-based constitutive modeling (Spencer 1984) to investigate tissue structure–function relationships. Commonly, in these studies, phenomenological strain energy density functions developed based on the model are curve fit to experimental data of bulk tissue mechanics to calibrate for model parameters that describe the structural contributions of tissue subcomponents and their interactions. The structure-based constitutive models have been valuable for highlighting the importance of fiber–matrix interactions with respect to degeneration and different loading conditions (Wu and Yao 1976; Klisch and Lotz 1999; Elliott and Setton 2001; Bass et al. 2004; Wagner and Lotz 2004; Yin and Elliott 2005; Peng et al. 2006; Wagner et al. 2006; Guerin and Elliott 2007; Nerurkar et al. 2008, 2011; O’Connell et al. 2009, 2012). However, these models often include a large number of hypothesized invariant terms, generating nonunique model parameters that cannot be easily compared or applied across studies (Yin and Elliott 2005; Guo et al. 2012). Directly linking model parameters to tissue physical properties and measurable tissue compositional changes has also been difficult as most parameters are not physically interpretable (Yin and Elliott 2005; Eskandari et al. 2019).

Additionally, the constitutive models normally performed poorly in simultaneously predicting tissue mechanics under multiple test configurations, due to the commonly applied model parameter calibration approach. Typically, the models are calibrated by curve fitting to study-specific stress–strain curves, often from a single test configuration (Sun et al. 2005; Schmidt et al. 2006, 2007), resulting in a limited model accuracy and robustness under other loading modalities. For example, previous work showed that constitutive models calibrated to uniaxial tension data were not able to accurately predict mechanical behaviors under biaxial tension or simple shear (Bass et al. 2004; O’Connell et al. 2012). Simultaneous curve fitting to multiple loading modalities has also proved challenging, often resulting in relatively poor model fits (Klisch and Lotz 1999; Wagner et al. 2006).

To address some of these issues, there has been a growing interest in using finite element models (FEM) to study three-dimensional tissue deformations. So far, most bulk tissue-scale FEMs employ homogenization theory, where every model element includes a combined and homogenized description of tissue subcomponents (e.g., fibers and the matrix) (Bensoussan et al. 1978; Sanchez-Palencia and Zaoui 1987; Jones 1999; Yin and Elliott 2005). This approach has allowed researchers to study three-dimensional stress and strain distributions, which has been valuable for predicting peak strains at failure (Eberlein et al. 2001) and for directing experimental protocol designs (Jacobs et al. 2013; Werbner et al. 2017). Unfortunately, homogenization of tissue subcomponents does not accurately represent the heterogeneous architecture of native tissues, where fibers and the extrafibrillar matrix are distinct materials that occupy separate volumes. Therefore, these models are not capable of describing and explaining some recent experimental observations, including variations in collagen fibril diameter with osmotic loading and changes in interfibrillar strain field with mechanical loading (Han et al. 2012; Vergari et al. 2016).

To address the limitations of the discussed modeling approaches, the objective of this study was to develop and validate a structure-based FEM that can be used to investigate multiscale structure–function relationships of fiber-reinforced tissues. To do so, we developed a model based on the native heterogeneous structure of the human AF, where fibers and the extrafibrillar matrix were described as two distinct materials occupying separate volumes (SEP model). The model was calibrated and validated using a multiscale framework. Model parameters were calibrated to sub-tissue-scale mechanical test data (Holzapfel et al. 2005), while model was validated at the bulk scale by comparing model-predicted multiaxial mechanics of multi-lamellar structures with multi-lamellar experimental test data. Multi-lamellar models developed using homogenization theory (HOM models) were also created, and their validation results were compared to results from the multiscale structure-based models. The second objective of this study was to investigate the relationship between specimen geometry and bulk tissue mechanics using the validated multiscale structure-based model. Although this study was conducted using AF morphology, the approaches and techniques employed here are applicable to other fiber-reinforced biological tissues and composites.

## Methods

### Model development

**Fig. 1:**
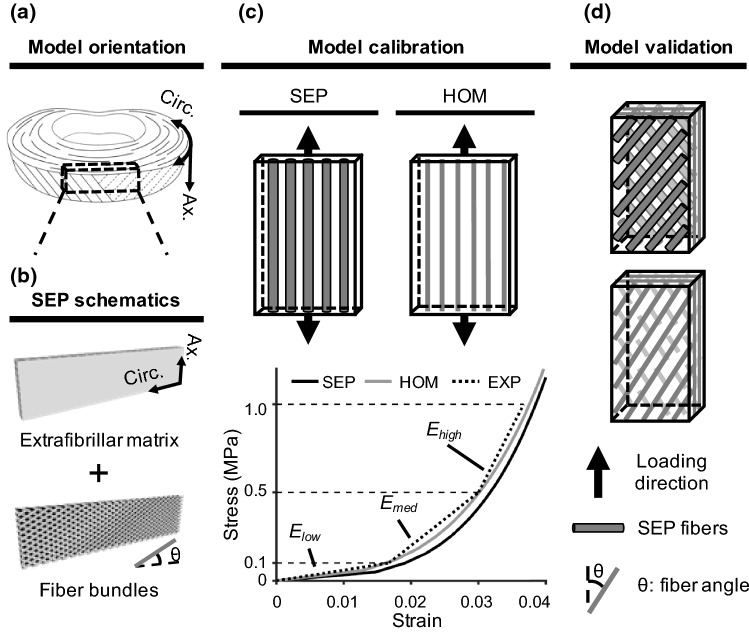
**a** Schematic of model orientation (circumferential: circ.; axial: ax.). **b** Separate model (SEP) described the extrafibrillar matrix and fiber bundles as two distinct materials that occupied separate volumes. **c** Single-lamellar models were used for model parameter calibration to experimental data (EXP) in the low-, medium-, and high-stress regions of the stress–strain curve (*E*_low_, *E*_med_, and *E*_high_, respectively) (Holzapfel et al. 2005). **d** After model calibration, multi-lamellar models were developed for validation. Bulk tissue mechanical properties were predicted and compared to data in the literature

Finite element models were developed with geometry and dimensions representative of specimens used in uniaxial tensile testing of the AF (SolidWorks 2017; Abaqus 6.14; ANSA 15.2.0; PreView 1.19.0; and FEBio 2.5.2, ~ 0.5–1 million tetrahedral elements, depending on specimen geometry). Each lamella had a thickness of 0.2 mm, based on native tissue properties (Marchand and Ahmed 1990). Previous experimental data suggested that AF modulus can change with specimen thickness (Żak and Pezowicz 2013, 2016). Thus, preliminary work was performed to determine whether specimen thickness, determined by the number of lamellae included in the model, affected bulk tissue modulus. To do this, a series of FEMs with identical specimen length and width but different thicknesses (i.e., number of lamellae) were developed to represent uniaxial tensile testing specimens along the axial direction (Fig. 1a).

A structure-based approach was employed during SEP model development to describe the AF as a fiber-reinforced composite containing distinct materials for the extrafibrillar matrix (matrix) and fiber bundles (SEP for “separate model”; Fig. 1b). Fiber bundles (fibers) were described as being uniformly distributed, full-length cylinders welded to the surrounding matrix (Shirazi-Adl et al. 1984; Goel et al. 1995; Michalek et al. 2009; Schollum et al. 2010). The radius of each fiber bundle was 0.06 mm, and interfibrillar spacing within each lamella was 0.22 mm (Marchand and Ahmed 1990). Fiber bundles were oriented at ± 30° (Fig. 1b—$\theta = 30^\circ$) to the transverse plane to represent specimens prepared from the middle-outer AF (Cassidy et al. 1989).

Triphasic mixture theory was employed to describe swelling in both SEP and HOM models to account for tissue hydration (Lai et al. 1991; Ateshian et al. 2004). Tissue permeability (*k*) was described as being strain-dependent (Holmes–Mow description; Eq. 1):

1 $$k\left( J \right) = k_{0} \left( {\frac{{J - \varphi_{0} }}{{1 - \varphi_{0} }}} \right)^{\alpha } {\text{e}}^{{\frac{1}{2}M\left( {J^{2} - 1} \right)}}$$

In Eq. 1, $$J$$ is the determinant of the deformation gradient tensor (***F***), $$k_{0}$$ is represented hydraulic permeability in the reference state ($$k_{0} = 0.0064$$ mm^4^/N s), $$\varphi_{0}$$ represents the solid volume fraction ($$\varphi_{0} = 0.3$$), $$\alpha$$ represents the power-law exponent ($$\alpha = 2$$), and $$M$$ represents exponential strain-dependence coefficient ($$M = 4.8$$) (Mow et al. 1984; Antoniou et al. 1996; Iatridis et al. 1998; Gu et al. 1999; Beckstein et al. 2008; Cortes et al. 2014; O’Connell et al. 2015). Fixed charge density, which represents the tissue proteoglycan content and drives tissue swelling, was set to -100 mmol/L for the matrix (middle-outer AF) and 0 mmol/L for fibers (i.e., no active swelling in the fibers) (Urban and Maroudas 1979; Huyghe et al. 2003). The osmotic coefficient (0.927) was determined using a linear interpolation of the data reported in Robinson and Stokes (1949) and Partanen et al. (2017). Free diffusivity ($$D_{0} )$$ and AF tissue diffusivity (*D*_AF_) of Na^+^ and Cl^−^ were set based on data in Gu et al. (2004), and 100% ion solubility was assumed ($$D_{{0,{\text{Na}}^{ + } }} = 0.00116$$ mm^2^/s; $$D_{{0,{\text{Cl}}^{ - } }} = 0.00161$$ mm^2^/s; $$D_{{{\text{AF}},{\text{Na}}^{ + } }} = 0.00044$$ mm^2^/s; $$D_{{{\text{AF}},{\text{Cl}}^{ - } }} = 0.00069$$ mm^2^/s).

For SEP models, the matrix was modeled as a compressible hyperelastic material using the neo-Hookean description (Bonet and Wood 1997) (Eq. 2), where $I_{1}$ and $I_{2}$ are the first and second invariants of the right Cauchy–Green deformation tensor, $\varvec{C }\left( {\varvec{C} = \varvec{F}^{\text{T}} \varvec{F}} \right)$ (Maas et al. 2012). $E_{\text{matrix}}$ and $\nu_{\text{matrix}}$ represent the Young’s modulus and Poisson’s ratio of the matrix. Fiber bundles in SEP models were described as a compressible hyperelastic ground matrix substance reinforced by power-linear fibers. The ground matrix substance was described using the Holmes–Mow material description, where $I_{1}$, $I_{2}$, $J$, $E_{\text{matrix}}$ and $\nu_{\text{matrix}}$ are defined as described above and $\beta$ represents the exponential stiffening coefficient (Eqs. 3–5) (Holmes and Mow 1990; Maas et al. 2012). The power-linear fiber description described AF nonlinearity and anisotropy, where $\gamma$ represents the power-law exponent in the toe region, $E_{{{\text{lin}}.}}$ represents the fiber modulus in the linear region and $\lambda_{0}$ represents the transition stretch between the toe and linear region (Eq. 6). Parameter $B$ is described as a function of $\gamma$, $E_{{{\text{lin}}.}}$, and $\lambda_{0}$ ($B = \frac{{E_{{{\text{lin}}.}} }}{2}\left( {\frac{{\left( {\lambda_{0}^{2} - 1} \right)}}{{2\left( {\gamma - 1} \right)}} + \lambda_{0}^{2} } \right)$). Lastly, fibers were described as being active only in tension:

2 $$W_{\text{matrix}} \left( {I_{1} ,I_{2} ,J} \right) = \frac{{E_{\text{matrix}} }}{{4\left( {1 + \nu_{\text{matrix}} } \right)}}\left( {I_{1} - 3} \right) - \frac{{E_{\text{matrix}} }}{{2\left( {1 + \nu_{\text{matrix}} } \right)}}\ln J + \frac{{E_{\text{matrix}} \nu_{\text{matrix}} }}{{\left( {1 + \nu_{\text{matrix}} } \right)\left( {1 - 2\nu_{\text{matrix}} } \right)}}\left( {\ln J} \right)^{2}$$

3 $$W_{\text{fiber}} \left( {I_{1} ,I_{2} ,J} \right) = \frac{1}{2}c\left( {e^{Q} - 1} \right)$$

4 $$\begin{aligned} Q & = \frac{{\beta \left( {1 + \nu_{\text{matrix}} } \right)\left( {1 - 2\nu_{\text{matrix}} } \right)}}{{E_{\text{matrix}} \left( {1 - \nu_{\text{matrix}} } \right)}}\left[ {\left( {\frac{{E_{\text{matrix}} }}{{1 + \nu_{\text{matrix}} }} - \frac{{E_{\text{matrix}} \nu_{\text{matrix}} }}{{\left( {1 + \nu_{\text{matrix}} } \right)\left( {1 - 2\nu_{\text{matrix}} } \right)}}} \right)\left( {I_{1} - 3} \right)} \right. \\ & \quad + \,\frac{{E_{\text{matrix}} \nu_{\text{matrix}} }}{{\left( {1 + \nu_{\text{matrix}} } \right)\left( {1 - 2\nu_{\text{matrix}} } \right)}}\left( {I_{2} - 3} \right) \\ & \quad \left. { - \,\left( {\frac{{E_{\text{matrix}} }}{{1 + \nu_{\text{matrix}} }} + \frac{{E_{\text{matrix}} \nu_{\text{matrix}} }}{{\left( {1 + \nu_{\text{matrix}} } \right)\left( {1 - 2\nu_{\text{matrix}} } \right)}}} \right)\ln J^{2} } \right] \\ \end{aligned}$$

5 $$c = \frac{{E_{\text{matrix}} \left( {1 - \nu_{\text{matrix}} } \right)}}{{2\beta \left( {1 + \nu_{\text{matrix}} } \right)\left( {1 - 2\nu_{\text{matrix}} } \right)}}$$

6 $$\psi_{n} \left( {\lambda_{n} } \right) = \left\{ {\begin{array}{*{20}l} 0 \hfill & {\lambda_{n} < 1} \hfill \\ {\frac{{E_{{{\text{lin}} .}} }}{{4\gamma \left( {\gamma - 1} \right)}}\left( {\lambda_{0}^{2} - 1} \right)^{2 - \gamma } \left( {\lambda_{n} - 1} \right)^{\gamma } } \hfill & {1 \le \lambda_{n} \le \lambda_{0} } \hfill \\ {E_{{{\text{lin}} .}} \left( {\lambda_{n} - \lambda_{0} } \right) + B\left( {\lambda_{n}^{2} - \lambda_{0}^{2} } \right) + \frac{{E_{{{\text{lin}} .}} }}{{4\gamma \left( {\gamma - 1} \right)}}\left( {\lambda_{0}^{2} - 1} \right)^{2 - \gamma } \left( {\lambda_{n} - 1} \right)^{\gamma } } \hfill & {\lambda_{n} > \lambda_{0} } \hfill \\ \end{array} } \right.$$

For FEMs that employed homogenization theory (HOM), a compressible hyperelastic Holmes–Mow material description was used to describe the ground matrix substance. Similar to SEP models, AF nonlinearity and anisotropy were incorporated by embedding a fiber description within the matrix. Fibers were described using a power-linear stress–strain relationship. Strain energy density functions for the ground matrix substance and fibers were identical to those used in the SEP models (Eqs. 3–6).

### Multiscale model calibration and validation framework

A multiscale framework was applied during model calibration and validation. First, single-lamellar SEP and HOM models were developed, and model parameters were calibrated to experimental data from single-lamellar uniaxial tensile tests both along and transverse to the fiber direction (experimental data from ventrolateral external AF; Fig. 1c) (Holzapfel et al. 2005). Model calibration was conducted until the computational Young’s modulus for both model types in the low-, medium-, and high-stress regions was within 10% of experimental data (Fig. 1c—stress–strain curves; Table 1). Calibrated model parameters that can be directly linked to tissue physical properties were also compared to data in the literature. Then, model validation was performed by predicting multiaxial bulk tissue mechanics using multi-lamellar specimens (Fig. 1d). For each SEP model, an HOM model with identical specimen length, width, and thickness was developed. As the more commonly used modeling approach, the validation results of the HOM models were considered as a baseline for comparison with SEP models.

**Table 1:** Young’s modulus obtained from SEP and HOM model calibration compared to experimental data [EXP, average (standard deviation)]. Experimental data taken from Holzapfel et al. (2005)

	EXP	SEP	HOM
*E*_low_ (MPa)	5.96(3.05)	5.7	5.9
*E*_med_ (MPa)	32.5(12.1)	32.0	30.3
*E*_high_ (MPa)	77.6(20.0)	74.6	70.0

**Fig. 2:**
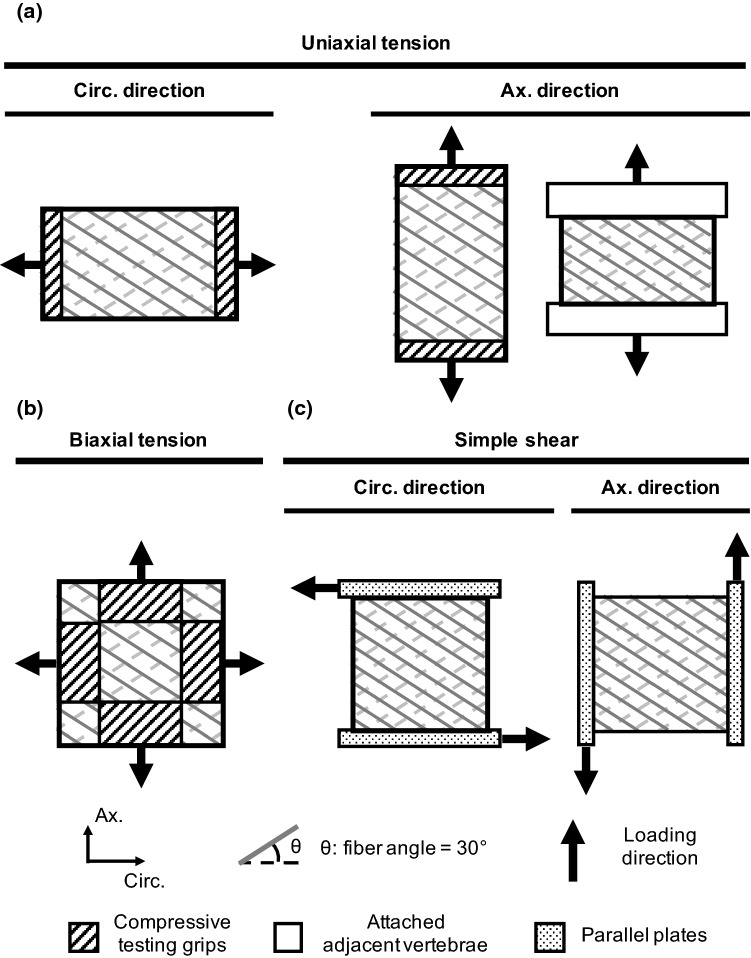
Schematics of evaluated loading modalities and boundary conditions used for multi-lamellar model validation. Model-predicted moduli from **a** uniaxial tension, **b** biaxial tension, and **c** simple shear were compared to data in the literature (*n* = 13 cases)

Model robustness was evaluated by simulating a range of reported loading modalities and boundary conditions. Simulated loading modalities included uniaxial tension along the circumferential and axial directions (Fig. 2a), biaxial tension in the circumferential–axial plane (Fig. 2b), and simple shear along the circumferential and axial directions (Fig. 2c). Three boundary conditions were evaluated, based on differences in reported gripping methods (*gripped*, *vertebrae*-*attached*, and *parallel*-*plate*, Fig. 2). The gripped boundary condition represented sandpaper glued to specimens and used to interface with testing equipment (Acaroglu et al. 1995; Elliott and Setton 2001; Guerin and Elliott 2006; O’Connell et al. 2009, 2012). The vertebrae-attached boundary condition referred to the case where tissue testing was prepared with the adjacent vertebrae attached to the AF and used to interface with test equipment (Green et al. 1993; Żak and Pezowicz 2016). The parallel-plate boundary condition described the case where specimens were clamped between polystyrene parallel plates for simple shear testing (Fujita et al. 2000).

Each validation model was loaded in a two-step process. Free swelling in 0.15 M phosphate-buffered saline was simulated prior to mechanical loading to account for specimen hydration. For uniaxial tension, a 20% engineering strain was applied. For biaxial tension, corresponding strain was applied in the circumferential and axial directions to represent the relative strain ratios reported in the literature [circumferential/axial strain ratios = 1:1 (equibiaxial) and 1:0 (axial-fixed)]. The simulation for biaxial tension was terminated when strain in either direction reached 15%. For simple shear, a 10% shear strain was applied in either circumferential or axial direction. Linear-region, apparent, or shear modulus was calculated as the slope of the corresponding stress–strain curve in the linear region and compared to values reported in the literature. Valid SEP or HOM model predictions of multi-lamellar mechanics were defined as predicted bulk modulus being within one standard deviation of reported mean values (Green et al. 1993; Acaroglu et al. 1995; Fujita et al. 2000; Elliott and Setton 2001; Guerin and Elliott 2006; O’Connell et al. 2009; Żak and Pezowicz 2016).

For a more rigorous validation, an exhaustive set of literature data was included for each loading modality and boundary condition (Table 2). Studies that conducted tissue-level tests using multi-lamellar specimens obtained from anterior middle-outer healthy human AF qualified for validation tests as long as relevant experimental protocols including tissue hydration, specimen orientation, and boundary and loading condition applied, were explicitly reported. Data from Green et al. 1993 were included despite the relatively high strain rate used, because it has been observed that modulus was not rate dependent when low to medium strain rates were applied (i.e., < 0.5 s^−1^) (Green et al. 1993; Kasra et al. 2004). Mean and standard deviations for moduli were pooled across studies by calculating the weighted average of mean or standard deviations ($E_{\text{pooled}} = \frac{{\mathop \sum \nolimits_{i = 1}^{s} n_{i} E_{i} }}{{\mathop \sum \nolimits_{i = 1}^{s} n_{i} }}$, ${\text{SD}}_{\text{pooled}} = \frac{{\sqrt {\mathop \sum \nolimits_{i = 1}^{s} n_{i} {\text{SD}}_{i}^{2} } }}{{\mathop \sum \nolimits_{i = 1}^{s} n_{i} - s}}$, where *s* represents total number of studies included, *n* represents study-specific sample size, and *E*_i_ and SD_*i*_ represent the mean and standard deviation of the modulus reported in each study).

**Table 2:** Summary of experimental data used for model validation, including sample size (*n*), tested specimen orientation, testing boundary condition, loading rate, reported modulus, and linearity of multi-lamellar stress–strain response (*NL* nonlinear; *PL* pseudo-linear). Bulk tissue mechanics reported as [average (standard deviation)] (*N.P.* not provided in study)

	Uniaxial tension						Biaxial tension	Simple shear
	Green et al. (1993)	Acaroglu et al. (1995)	Elliott and Setton (2001)	Guerin and Elliott (2006)	O’Connell et al. (2009)	Zak and Pezowicz (2016)	O’Connell et al. (2012)	Fujita et al. (2000)
*n*	9	15	Ax.: 12; circ.: 20	8	7	18	16	20
Orientation tested	Ax.	Circ.	Ax.; circ.	Circ.	Ax.; circ.	Ax.	Ax.-circ. plane	Ax.; circ.
Boundary condition	Vertebrae-attached	Gripped	Gripped	Gripped	Gripped	Vertebrae-attached	Gripped	Parallel-plate
Loading rate	4 mm/s	0.0001 s^−1^	0.0001 s^−1^	0.0001 s^−1^	0.0001 s^−1^	0.5 mm/s	0.0001	N.P.
Modulus (MPa)	Linear_circ._: 16.4 (7.0)	Linear_circ._: 27.0 (15.0)	Toe_ax._: 0.27(0.28) Linear_ax._: 0.82(0.71) Toe_circ._: 2.52(2.27) Linear_circ._: 17.45(14.29)	Toe_circ._: 2.53(1.47) Linear_circ._: 29.35(21.92)	Linear_ax._: 0.42(0.11) Toe_circ._: 2.70 (2.33) Linear_circ._: 20.90 (13.50)	Linear_ax._: 21.96(12.77)	N.P.	Shear_ax._: 0.22(0.11) Shear_circ._: 0.11(0.06)
Linearity	NL	NL	Ax.: PL; circ.: NL	NL	Ax.: PL; circ.: NL	NL	NL	N.P.

### Effect of specimen geometry on tensile mechanics

Following model validation, the effect of specimen geometry on AF bulk mechanics was investigated, because experimental observations noted that modulus was sensitive to specimen geometry (Adams and Green 1993; Lechner et al. 2000; Werbner et al. 2017). Additional uniaxial multi-lamellar SEP models were created along the circumferential direction (*n* = 50 models; Fig. 1a). Specimen geometry for length was varied between 6 and 15 mm in 1 mm increments, and width was varied between 2 and 3 mm in 0.25 mm increments, resulting in length-to-width aspect ratios (AR) between 2.0 and 7.5. Uniaxial tension was applied as described above, and the predicted linear-region modulus was calculated. During loading, specimen top and bottom surfaces were constrained to restrict displacement in the loading direction.

A multivariate linear regression model was used to characterize the relationship between bulk tissue modulus (*y*) and specimen geometry ($x_{1}$: length; $x_{2}$: 1/width; Eq. 7; R software, Foundation for Statistical Computing, Vienna, Austria). In Eq. 7, $\beta_{i}$ represent regression parameters, which were determined using the least squares method, and $\varepsilon$ represents errors in the statistical model. The effect of specimen width was represented as 1/width to incorporate aspect ratio as an interaction term (i.e., length/width or $x_{1} x_{2}$). If a parameter, $\beta_{i}$, was determined to be statistically insignificant, it was removed from the model and the analysis was repeated with the reduced linear regression model. Significance was assumed for *p* values ≤ 0.05. The relative contribution of specimen length, width, and aspect ratio to AF tensile modulus was calculated using the relaimpo package and reported as a percent (Grömping 2006).

7 $$y = \beta_{0} + \beta_{1} x_{1} + \beta_{2} x_{2} + \beta_{3} x_{1} x_{2} + \varepsilon$$

## Results

### Multiscale model calibration and validation

**Fig. 3:**
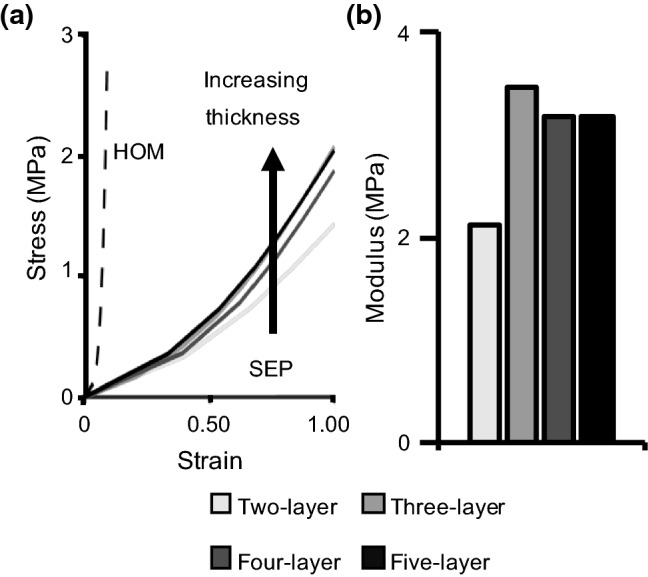
**a** Stress–strain response from SEP (solid lines) and HOM (dashed lines) models with two to five lamellae. Stress–strain curves for HOM models were identical, regardless of the specimen thickness. **b** Predicted linear-region modulus of two-, three-, four-, and five-layer SEP models

Our preliminary work showed that SEP model-predicted bulk tissue modulus was consistent for models with three or more lamellae (Fig. 3), while HOM model-predicted bulk tissue modulus was not affected by specimen thickness (Fig. 3a—overlapping dashed lines). Based on these findings, multi-lamellar models of both model types were developed with three layers for computational efficiency.

**Fig. 4:**
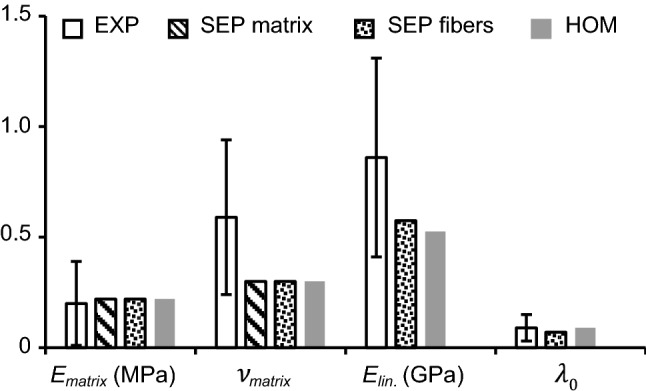
Calibrated SEP and HOM model parameters compared to experimental (EXP) values. Experimental data taken from Fujita et al. (1997), Elliott and Setton (2001), Holzapfel et al. (2005), Van der Rijt et al. (2006), Shen et al. (2008), O’Connell et al. (2009), and Cao et al. (2009)

Stress–strain curves from calibrated single-lamellar HOM and SEP models were nonlinear, agreeing well with the literature (Fig. 1c). For both model types, computational modulus for the low-, medium-, and high-stress regions of the stress–strain curve also matched values in the literature (Table 1). Calibrated model parameters for both model types are summarized in Table 3; parameters that can be linked to tissue physical properties also had values that agreed well with reported values (Table 3; Fig. 4—parameter values were within one standard deviation of reported means).

**Table 3:** Summary of calibrated model parameters for SEP and HOM models. Experimental data from sub-tissue mechanical tests are reported as [average (standard deviation)]. Experimental data taken from Fujita et al. (1997), Elliott and Setton (2001), Holzapfel et al. (2005), Van der Rijt et al. (2006), Shen et al. (2008), O’Connell et al. (2009), and Cao et al. (2009) (*N.A.* not applicable)

	EXP	SEP		HOM
		Matrix	Fibers	
*E*_matrix_ (MPa)	0.2(0.19)	0.22	0.22	0.22
***ν***_matrix_	0.59(0.35)	0.3	0.3	0.3
*β*	N.A.	N.A.	1	1
*E*_lin._ (GPa)	0.86(0.45)	N.A.	0.58	0.53
***γ***	N.A.	N.A.	5.95	6
***λ***_0_	1.09(0.06)	N.A.	1.07	1.09

**Fig. 5:**
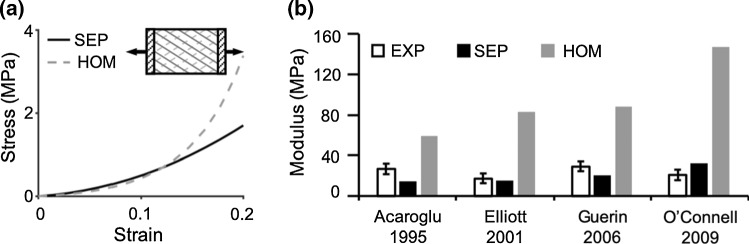
**a** Representative stress–strain response from SEP and HOM models under uniaxial tension (circumferential direction). **b** Model-predicted linear-region modulus compared to experimental (EXP) data

A summary of model validation results is provided in Table 4. Simulations of uniaxial tensile tests along the circumferential direction were all subjected to the gripped boundary condition (four models; Fig. 5a—inset). Multi-lamellar SEP and HOM models both demonstrated a nonlinear stress–strain response (Fig. 5a; Table 4—‘Lin.’). The circumferential-direction toe-region modulus was ~ 4 MPa for both SEP and HOM model types and was within one standard deviation of reported values (pooled experimental toe-region modulus = 2.6 ± 2.1 MPa) (Elliott and Setton 2001; Guerin and Elliott 2006; O’Connell et al. 2009). However, at greater strains, there was a large deviation in predicted behavior by SEP and HOM models (Fig. 5a). SEP-predicted linear-region modulus was within the range of reported values (< 0.9× standard deviation from the reported mean; Fig. 5b—white versus black bars) (Acaroglu et al. 1995; Elliott and Setton 2001; Guerin and Elliott 2006; O’Connell et al. 2009). In contrast, HOM models overestimated the linear-region modulus by 120–600% (> 2× standard deviations from the reported mean; Fig. 5b—white versus gray bars).

**Table 4:** SEP- and HOM-predicted linearity of multi-lamellar tissue stress–strain behavior (*Lin.* linearity; *NL* nonlinear; and *PL* pseudo-linear). Model-predicted moduli (mod.) were compared to pooled (if applicable) experimental (EXP) data in the literature [average (standard deviation)]

	Uniaxial tension							Biaxial tension						Simple shear	
	Circ. (gripped)			Ax. (gripped)		Ax. (vertebrae-attached)		Equibiax., circ. (gripped)		Equibiax., ax. (gripped)		Ax.-fixed, circ. (gripped)		Circ. (parallel-plate)	Ax. (parallel-plate)
	Lin.	Toe mod. (MPa)	Linear mod. (MPa)	Lin.	Mod. (MPa)	Lin.	Mod. (MPa)	Lin.	Apparent mod. (MPa)	Lin.	Apparent mod. (MPa)	Lin.	Apparent mod. (MPa)	Shear mod. (kPa)	Shear mod. (kPa)
EXP	NL	2.52(2.08)	21.10(15.80)	PL	0.67(0.57)	NL	20.11(11.25)	NL	~ 60	NL	~ 30	NL	~ 40	111(56)	224(112)
SEP	NL	4.70	23.0	PL	0.60	NL	18.6	NL	58	NL	21	NL	31	163	1375
HOM	NL	3.20	106.2	PL	0.70	NL	59.7	PL	33	PL	7	PL	16	326	1697

**Fig. 6:**
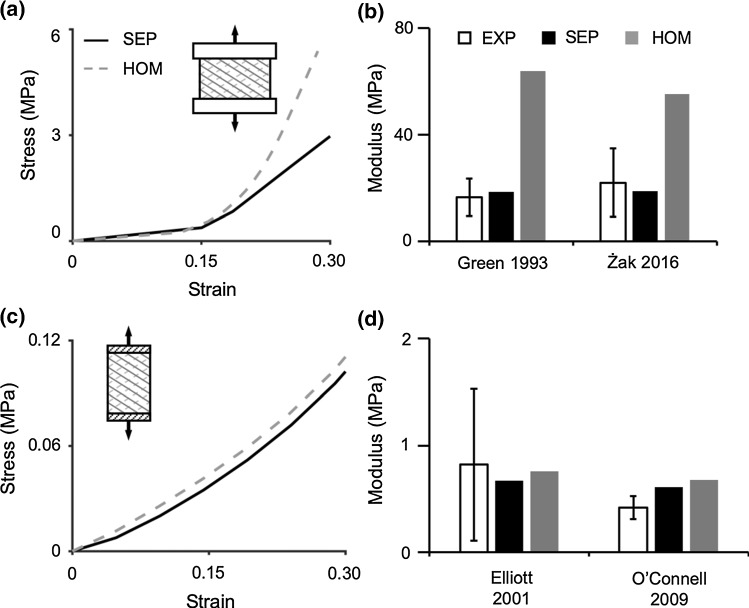
**a**, **c** Representative stress–strain response from SEP and HOM models under uniaxial tension (axial direction). Evaluated boundary conditions included **a** vertebrae-attached and **c** gripped. Model-predicted linear-region modulus compared to corresponding experimental (EXP) data that used **b** vertebrae-attached or **d** gripped boundary conditions

Four model simulations were performed to evaluate SEP and HOM models response under uniaxial tension in the axial direction (Fig. 6). Two model simulations were subjected to the vertebrae-attached boundary condition (Fig. 6a—inset), and two model simulations were subjected to the gripped boundary condition (Fig. 6c—inset). For the vertebrae-attached specimens, multi-lamellar SEP and HOM models both demonstrated a nonlinear stress–strain response (Fig. 6a). Similar to results for uniaxial tension along the circumferential direction, SEP and HOM model predictions for toe-region modulus were comparable to each other and agreed with data in the literature (~ 2.5 MPa), while differences in tissue mechanics predicted by the two model types were more pronounced at larger strains (i.e., HOM models predicted greater stresses in the linear region). SEP-predicted linear-region modulus was within 15% of the reported mean value (< 0.26× standard deviation away from the reported mean; Fig. 6b). However, HOM models predicted a linear-region modulus that was at least 150% greater than reported values (> 2.5× standard deviation away from the reported mean; Fig. 6b) (Green et al. 1993; Żak and Pezowicz 2016). For the gripped specimens, SEP and HOM models both generated a similar pseudo-linear stress–strain curve (Fig. 6c) and accurately predicted the tensile modulus reported by Elliott and Setton (2001) (< 0.2× standard deviation from the reported mean; Fig. 6d). Model validation to data reported in O’Connell et al. (2009) resulted in an overestimation of the axial-direction tensile modulus, but the predicted modulus from both model types was on the same order of magnitude as the reported mean (SEP: overestimated modulus by ~ 45% or 1.7× standard deviations from the reported mean, HOM: overestimated modulus by ~ 60% or 2.4× standard deviations from the reported mean; Fig. 6d) (O’Connell et al. 2009).

**Fig. 7:**
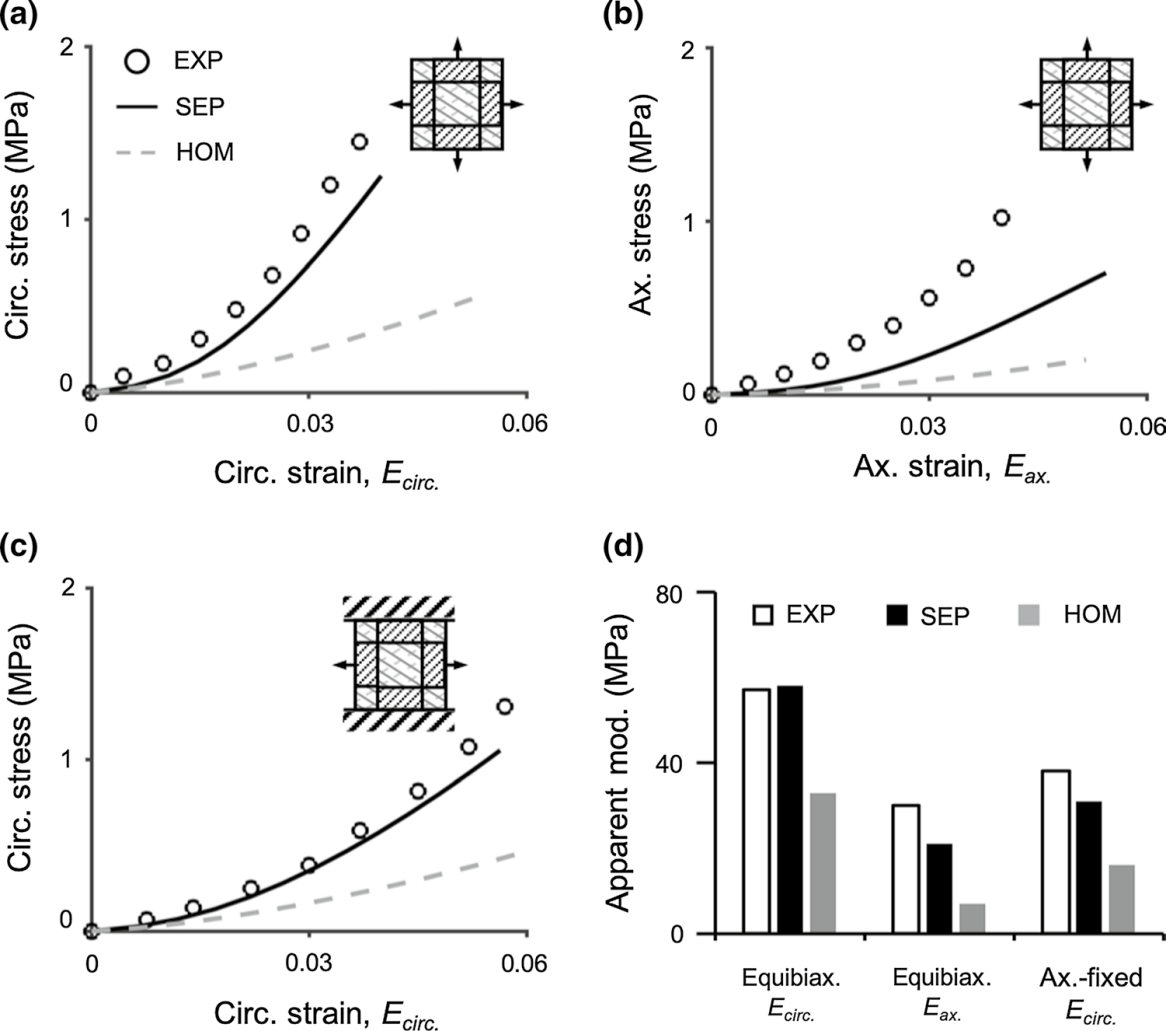
Stress–strain response from SEP and HOM models in the **a** circumferential and **b** axial directions under equibiaxial (equibiax.) tension. **c** Circumferential-direction stress–strain response from SEP and HOM models under the axial-fixed (ax.-fixed) loading condition. **d** Model-predicted apparent modulus compared to experimental data (EXP) reported in O’Connell et al. (2012)

Three model simulations were performed to evaluate SEP and HOM models response under biaxial tension. All model simulations were subjected to the gripped boundary condition (Fig. 7a to c—inset). Experimental average and standard deviation for apparent modulus were not reported; therefore, validations were performed using the representative stress–strain curve reported by O’Connell et al. (2012). In all validation cases, SEP and HOM models demonstrated a nonlinear and pseudo-linear stress–strain behavior, respectively (Fig. 7 a to c—black solid versus grey dashed curves). Under equibiaxial tension, SEP models accurately predicted the apparent modulus while HOM models underestimated the apparent modulus by ~ 45% in the circumferential direction (Fig. 7d—Equibiax., *E*_circ._); in the axial direction, SEP and HOM models underestimated the apparent modulus by ~ 30% and ~ 70%, respectively (Fig. 7d—Equibiax., *E*_ax._). Under the axial-fixed condition, SEP and HOM models underestimated the circumferential-direction apparent modulus by ~ 20% and ~ 60%, respectively (Fig. 7d—Ax.-fixed, *E*_circ._).

**Fig. 8:**
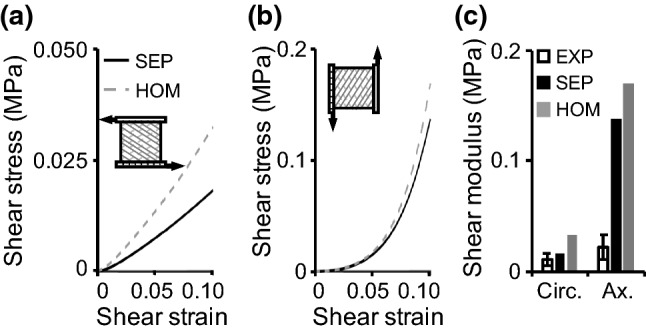
Stress–strain response from SEP and HOM models for simple shear in the **a** circumferential and **b** axial directions. **c** Model-predicted circumferential (circ.) and axial (ax.) shear modulus compared to experimental (EXP) values

Two model simulations were performed to evaluate SEP and HOM models response under simple shear. Both model simulations were subjected to the parallel-plate boundary condition (Fig. 8a, b—inset). In the circumferential direction, SEP and HOM models both predicted a pseudo-linear stress–strain response (Fig. 8a). The SEP-predicted shear modulus was ~ 160 kPa and matched well with reported values (< 0.93× standard deviation from the reported mean), while the HOM-predicted modulus was greater than 300 kPa or more than 200% greater than the reported mean (> 3.8× standard deviations from the reported mean; Fig. 8c) (Fujita et al. 2000). In the axial direction, SEP and HOM models both predicted a nonlinear stress–strain response (Fig. 8b), and both models greatly overestimated the axial-direction shear modulus (SEP: overestimated modulus by ~ 500% or > 10× standard deviations from the reported mean; HOM: overestimated by ~ 660% or > 13× standard deviations from the reported mean; Fig. 8c) (Fujita et al. 2000).

### Effect of specimen geometry on tensile modulus

**Fig. 9:**
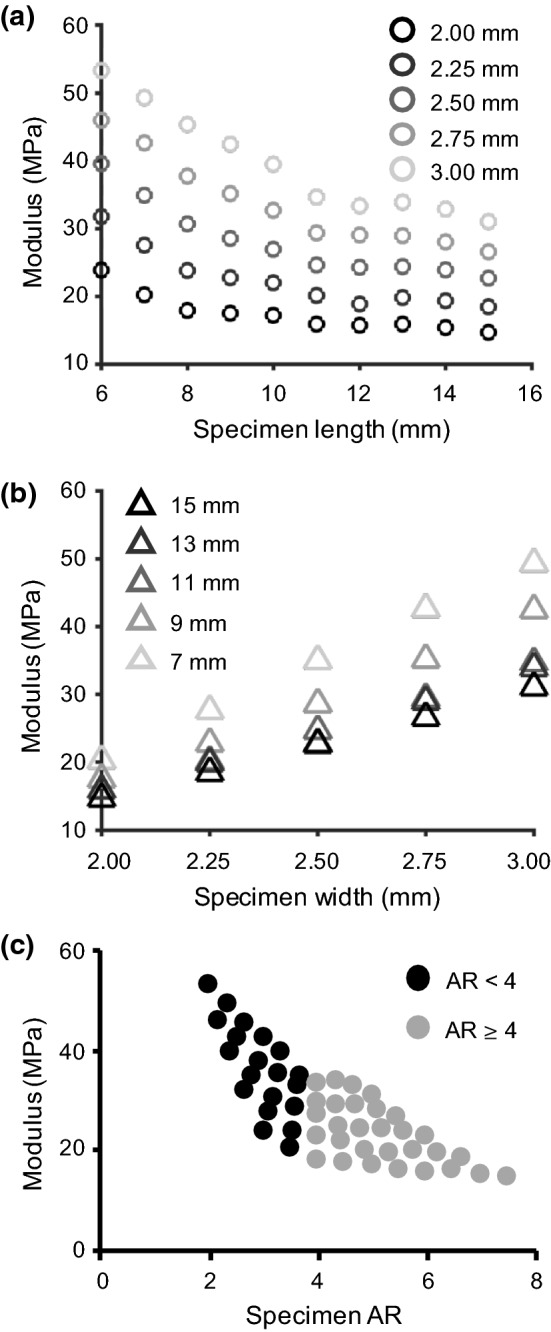
**a** Model-predicted tensile modulus with respect to specimen length for five specimen widths. **b** Modulus with respect to specimen width for five specimen lengths (specimens with even-value lengths followed a similar trend but were omitted in the figure for clarity); **c** Modulus with respect to specimen aspect ratio (AR)

After validation, the SEP model was used to study the effect of specimen geometry on bulk tissue modulus. A nonlinear decrease in AF tensile modulus was observed with an increase in specimen length (Fig. 9a). Based on this response, a logarithmic transformation was performed to determine the relationship between specimen geometry and bulk modulus with a multivariate linear regression. AF tensile modulus increased linearly with specimen width, and the rate of change in tensile modulus with specimen width was dependent on specimen length (Fig. 9b—slope_width = 7 mm_ ≈ 1.8 × slope_width = 15 mm_). This finding highlights the dependence of AF tensile modulus on the interaction between specimen length and width (i.e., aspect ratio), where tensile modulus decreased with an increase in aspect ratio (Fig. 9c). Moreover, it appeared that tensile modulus approached a horizontal asymptote as the aspect ratio exceeded 4.0 (ASTM guidelines for uniaxial test specimens (ASTM 2003, 2004); Fig. 9c—gray dots). Therefore, AF tensile modulus was a function of specimen length, width, and aspect ratio (Eq. 8; Supplementary Table). Lastly, based on the relative contribution analysis, AF tensile modulus was most sensitive to specimen width (48% contribution), followed by aspect ratio (36% contribution), and specimen length (16% contribution).

8 $$\log \left( {\text{modulus}} \right) = 3.4 - 1.1 \cdot \log \left( {\text{length}} \right) - 2.7 \cdot \left( {1/{\text{width}}} \right) + 0.06 \cdot {\text{AR}} + \varepsilon$$

## Discussion

It is common for model parameter calibrations to be performed at the same scale as the study of interest in both constitutive and finite element modeling studies. For example, to study bulk tissue mechanics, model calibration would be conducted based on multi-lamellar test data from a dataset obtained from a single loading modality, often limiting the model’s ability to accurately predict tissue mechanics under other loading modalities (Bass et al. 2004). Moreover, findings from this study and experimental observations suggest that this curve-fitting approach is limited to specimens with a specific geometry constrained by a particular boundary condition, further restricting the predictive power and the robustness of the model (Adams and Green 1993; Sun et al. 2005; Jacobs et al. 2013; Werbner et al. 2017). Additionally, while these models are widely used to understand contributions of sub-tissue properties to bulk tissue mechanics, it has been difficult to establish relationships between model parameters and tissue physical properties (e.g., collagen stiffness) or biochemical compositions (e.g., cross-links) as model parameters can be nonunique and are purely mathematical coefficients without physical significance (Yin and Elliott 2005; Eskandari et al. 2019).

To address these limitations, we employed a unique multiscale framework for model calibration and validation in this study. Specifically, for both SEP and HOM model types, we considered multi-lamellar AF as a superposition of individual lamellae, which represented the fundamental structural unit (Holzapfel et al. 2005). While model calibration was performed at the sub-tissue scale using single-lamellar experimental data, model validation was performed at bulk tissue scale by predicting multiaxial mechanics using multi-lamellar models. This more rigorous approach ensured the accuracy and robustness of the model, if validated, such that the SEP model can be used to investigate tissue-level mechanics under multiple loading configurations and to understand the role of sub-tissue properties on tissue-level mechanics. Additionally, when developing SEP models, individual AF lamellae were modeled structure-based using known anatomical measurements, resulting in multi-lamellar models with a fibrous network that better resembled the native tissue.

The parameter calibration of single-lamellar SEP and HOM models resulted in a similar stress–strain response with almost identical computational moduli, suggesting that SEP and HOM models may predict similar mechanical behaviors for multi-lamellar specimens if the two modeling approaches shared a comparable accuracy and robustness. However, the SEP model type was rigorously validated (i.e., accurately predicted bulk tissue stress–strain response and corresponding moduli) in ten of 13 validation cases (> 75% passing rate), while the HOM model type was only validated in one validation case, proving the SEP model as a more accurate and robust modeling approach. Although the SEP model slightly overestimated the uniaxial tensile modulus in the axial direction as reported in O’Connell et al. (2009), we considered the SEP model prediction as acceptable, due to the relatively small difference in absolute values (difference between model-predicted modulus and experimental data = 0.19 MPa). Additionally, since only one representative stress–strain curve could be used for each biaxial tension validation case, it is also possible that the SEP model may be acceptable for describing axial-direction mechanics under equibiaxial loading (prediction was within 30% of the reported data) (O’Connell et al. 2012). However, it should be noted that the SEP model greatly overestimated the axial-direction shear modulus, which may be due to fibers being described as continuous bundles, resulting in an increased tissue stiffness due to the immediate engagement of the fiber bundles that extended between the parallel plates after the applied loading (Szczesny et al. 2015, 2017).

Attributed to the multiscale calibration framework, the majority of SEP model parameters (six of eight parameters) could be directly linked to tissue physical properties. The parameters included modulus and Poisson’s ratio of the ground matrix substance (*E*_matrix_, *ν*_matrix_), collagen fiber modulus (*E*_lin._), and transition strain ($\lambda_{0}$). Additionally, all calibrated values agreed well with reported values (Table 2 and Fig. 4) (Fujita et al. 1997; Elliott and Setton 2001; Holzapfel et al. 2005; Van der Rijt et al. 2006; Shen et al. 2008; Cao et al. 2009; O’Connell et al. 2009). This suggests that the SEP model parameters represent intrinsic tissue properties, broadening the model’s ability to study the effect of degeneration, disease, or injury on tissue mechanics. Particularly, the effect of tissue degeneration and regeneration can be investigated by adjusting fixed charge density of the extrafibrillar matrix, which is indicative to tissue degeneration in native tissues or tissue growth in engineered constructs (Adams and Roughley 2006; Nerurkar et al. 2007). The effect of disease can be investigated by varying fiber modulus, which has been shown to increase with greater fiber cross-linking with diabetes (Li et al. 2013; Svensson et al. 2018). Lastly, the effect of injury, which has been found to be rate dependent, can be investigated by changing the computational loading rate (Wang et al. 2000; Kasra et al. 2004).

To further demonstrate the predictive power of the SEP model, we evaluated the relationship between specimen geometry and AF tensile modulus, based on experimental observations that reported modulus sensitivity to specimen width (Adams and Green 1993; Werbner et al. 2017). A multivariate linear regression model was used to characterize AF tensile modulus as a function of specimen geometry, where specimen length and width were investigated as main factors and aspect ratio was evaluated as an interaction term. The regression analysis suggested that AF tensile modulus was a function of specimen length, width, and aspect ratio. Specifically, AF tensile modulus increased with specimen width and decreased with specimen length and aspect ratio, with specimen width being the most dominant factor. Therefore, unlike traditional engineering materials, AF tensile modulus may not be considered an intrinsic material property due the composite heterogeneous structure of the tissue, and it may be necessary to account for differences in specimen geometry when comparing data across studies. Our findings also suggest that obtaining consistent bulk tissue properties along the circumferential direction may be possible by using specimens with large aspect ratios and a smaller width, which agrees with recent work on meniscus, tendons, and ligaments (Wren et al. 2001; Peloquin et al. 2016; Creechley et al. 2017). Interestingly, Adams and Green (1993) and Werbner et al. (2017) both observed an increase in modulus as the midlength width relative to the grip width decreased. While the midlength-to-grip width ratio was not varied in this study, differences in trends may be due to a difference in fiber engagement, which can be directly evaluated with the SEP model, but not the HOM model.

A few assumptions were made to simplify the current SEP model. First, the fiber network did not include fiber dispersion (Guo et al. 2012), potential fibers in the radial direction (Marchand and Ahmed 1990), variation in fiber diameter or length (Marchand and Ahmed 1990; Han et al. 2012), or fiber–fiber interactions (e.g., cross-links). Particularly, cross-links have been shown to play an important role in tissue subfailure and failure mechanics and will be included in future iterations of the model (Moore et al. 1996; Elliott and Setton 2001; Adams and Roughley 2006; Guerin and Elliott 2006; Provenzano and Vanderby 2006; Roeder et al. 2009; O’Connell et al. 2009; Isaacs et al. 2014). Second, the current model did not investigate different mechanisms for fiber–matrix interactions, which have been suggested to be important for stress distribution during loading (Bruehlmann et al. 2004; Szczesny et al. 2015, 2017; Vergari et al. 2016).

In this study, we developed and validated a multiscale structure-based finite element model that accurately and robustly predicted AF bulk tissue mechanics under multiple loading configurations. Modeling fibers and the extrafibrillar matrix as separate materials, based on the native tissue architecture, resulted in uniquely determined model parameters with physical interpretations. Applying a multiscale framework for model calibration and validation resulted in a rigorous validation process that ensured and improved model accuracy and robustness. In conclusion, the multiscale structure-based modeling approach allows for studies that simultaneously investigate tissue- and sub-tissue-scale mechanics, which will be important for studying multiscale tissue mechanics with degeneration, disease, and injury (Iatridis and Gwynn 2004; Iatridis et al. 2005).

## Electronic supplementary material

Below is the link to the electronic supplementary material.

Supplementary material 1 (DOCX 89 kb)
