# Supplementary material for: Multiscale composite model of fiber-reinforced tissues with direct representation of sub-tissue properties
Source: Biomech Model Mechanobiol. 2019 Nov 4;19(2):745–59. doi: 10.1007/s10237-019-01246-x (PMC7105449; doi:10.1007/s10237-019-01246-x)
Supplement: Supplementary file 1 — Supplementary material 1 (DOCX 89 kb) [file 10237_2019_1246_MOESM1_ESM.docx]

**Supplementary Table 1** Summary of parameters from multivariate linear regression analysis for AF tensile modulus (Equation 8). $\hat{\beta}$represents regression coefficient and SE represents the standard error of the coefficient
